# Antibody-Drug Conjugates Targeting the Human Epidermal Growth Factor Receptor Family in Cancers

**DOI:** 10.3389/fmolb.2022.847835

**Published:** 2022-02-28

**Authors:** Jinfeng Yu, Tong Fang, Chengyu Yun, Xue Liu, Xiaoqing Cai

**Affiliations:** School of Pharmaceutical Sciences, Sun Yat-sen University, Guangzhou, China

**Keywords:** antibody-drug conjugates, cancer targeted therapy, drug resistance, EGFR, HER family, HER2, HER3

## Abstract

Members of the human epidermal growth factor receptor (HER) family, which includes HER1 (also known as EGFR), HER2, HER3 and HER4, have played a central role in regulating cell proliferation, survival, differentiation and migration. The overexpression of the HER family has been recognized as one of the most common cellular dysregulation associated with a wide variety of tumor types. Antibody-drug conjugates (ADCs) represent a new and promising class of anticancer therapeutics that combine the cancer specificity of antibodies with cytotoxicity of chemotherapeutic drugs. Two HER2-directed ADCs, trastuzumane-emtansine (T-DM1) and trastuzumab-deruxtecan (DS-8201a), have been approved for HER2-positive metastatic breast cancer by the U.S. Food and Drug Administration (FDA) in 2013 and 2019, respectively. A third HER2-directed ADC, disitamab vedotin (RC48), has been approved for locally advanced or metastatic gastric or gastroesophageal junction cancer by the NMPA (National Medical Products Administration) of China in 2021. A total of 11 ADCs that target HER family receptors (EGFR, HER2 or HER3) are currently under clinical trials. In this review article, we summarize the three approved ADCs (T-DM1, DS-8201a and RC48), together with the investigational EGFR-directed ADCs (ABT-414, MRG003 and M1231), HER2-directed ADCs (SYD985, ARX-788, A166, MRG002, ALT-P7, GQ1001 and SBT6050) and HER3-directed ADC (U3-1402). Lastly, we discuss the major challenges associated with the development of ADCs, and highlight the possible future directions to tackle these challenges.

## Introduction

The epidermal growth factor receptor (HER) family of receptor tyrosine kinase has been known to play essential roles in regulating cell proliferation, survival, differentiation and migration (Wieduwilt and Moasser 2008). This receptor family consists of four receptor members, including EGFR (HER1 or ERBB1), HER2 (ERBB2), HER3 (ERBB3) and HER4 (ERBB4) (London and Gallo 2020). These four receptors share five similar structural elements: a N-terminal glycosylated extracellular domain, a hydrophobic transmembrane domain, and a short intracellular juxtamembrane segment, a tyrosine kinase domain, and a tyrosine-containing C-terminal tail (Wieduwilt and Moasser 2008; Santos et al., 2021). Specific ligands have been identified for the extracellular domain of EGFR, HER3 and HER4, whereas there have been no known ligands that bind HER2 (Schlessinger 2002). Upon ligand binding, the receptors undergo dimerization, either as homodimers or heterodimers, which consequently activates the intracellular tyrosine kinase domain, and leads to the phosphorylation of the C-terminal tail (Linggi and Carpenter 2006; Kumar et al., 2020; Santos et al., 2021). These autophosphorylation events in turn activate the downstream signaling pathways, including the phosphatidylinositol 3-kinase (PI3K)/Akt pathway, the Ras/Raf/mitogen-activated protein kinase (MAPK) pathway and the STAT pathways, which subsequently induce cell proliferation (Lowenstein et al., 1992; Batzer et al., 1994; Hallberg et al., 1994).

The overexpression of the HER family has been recognized as one of the most common cellular dysregulation associated with various tumor types (Yarden and Pines 2012; Kumar et al., 2020). EGFR and HER2 are overexpressed in many solid tumors, including lung, head and neck, breast, kidney, gastric, colon, pancreatic, ovary, prostate and bladder cancers. Among the four HER family members, only EGFR can induce tumor proliferation through homodimerization, whereas the homodimerization of HER2, HER3 or HER4 possess no oncogenic property (Cohen et al., 1996). Despite having no known ligand, HER2 induce an aggressive tumorigenic phenotype through dimerization with other EGFR members, such as EGFR and HER3. HER2 possesses a superior ability to form heterodimers, representing the preferred dimerization partner for all the HER receptors (Wu and Shih 2018). Similar to HER2, HER3 functions through forming active heterodimers with other HER members, mainly HER2 or EGFR (Yarden and Pines 2012; Littlefield et al., 2014; Kumar et al., 2020). HER3 plays a crucial role in cancer progression, and is a significant marker for poor overall survival with various solid tumors (Ocana et al., 2013). Among all HER members, HER4 is the least overexpressed receptor in human cancers. In contrast to other HER receptors, HER4 has been found to trigger apoptosis and reduce proliferation in cancer cells through antagonizing HER2 signaling activity (Naresh et al., 2008).

**FIGURE 1:**
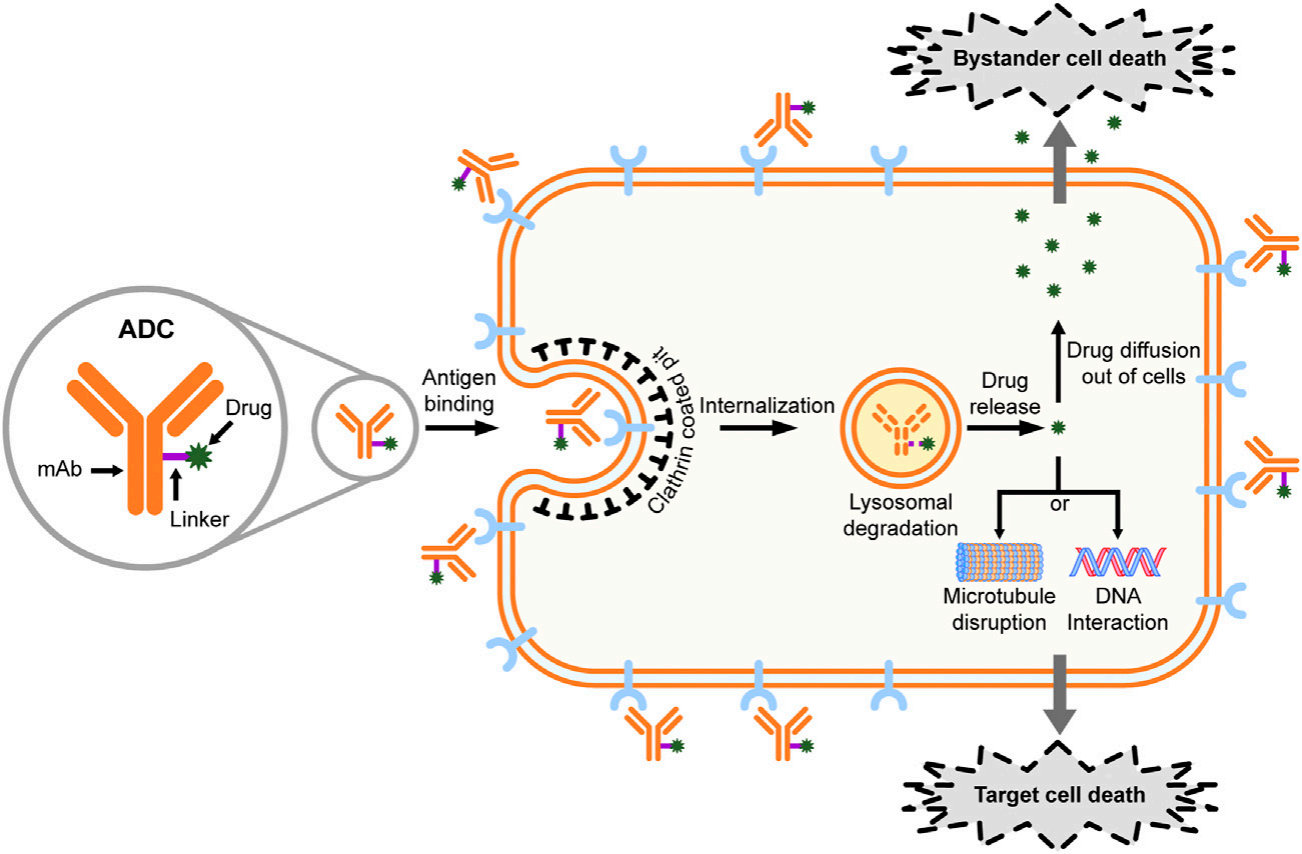
Structure and mechanism of action of antibody-drug conjugates (ADCs).

Antibody-drug conjugates (ADCs) represent a new and promising class of anticancer therapeutics that combine the cancer specificity of antibodies with cytotoxicity of chemotherapeutic drugs (Thomas et al., 2016; Abdollahpour-Alitappeh et al., 2019). Generally, an ADC is comprised of a target-specific monoclonal antibody (mAb) covalently linked to a cytotoxic drug with a chemically synthetic linker (Figure 1). The mAb components of ADCs bind to the specific antigen on the surface of cancer cells, leading to the internalizations of ADCs (Thomas et al., 2016). Ideally, ADCs are packed into vesicles upon internalization, followed by further transporting to lysosomes, where the acidic and proteolytic environment causes the release of the toxic compounds (Jin et al., 2021). In addition to the targeted cancer cells, the non-targeted cancer cells within the close proximity may also be killed by the “bystander effect” of ADCs (Kovtun et al., 2006; Bargh et al., 2019). Such bystander killing is generally expected to improve the efficacy of ADCs, although concerns have also been raised about the potential toxicity to normal cells (Chari et al., 2014).

As of December 2021, a total of 12 ADCs have been approved for the treatment of various cancers, and over 100 ADCs are currently under clinical trials. Herein, we review the three approved anti-HER2 ADCs and 11 investigational ADCs that target HER family receptors, including EGFR, HER2 and HER3. Lastly, we discuss the major challenges associated with the development of ADCs, and highlight the possible future approaches to tackle these challenges.

### mAbs and Tyrosine Kinase Inhibitors Targeting Human Epidermal Growth Factor Receptors

Due to the critical roles of HER1–3 in carcinogenesis, two main targeted therapies have been developed in the past two decades to block the HER-driven pathways, which include small molecule compounds that inhibit the tyrosine kinase activity of the intracellular domain, and mAbs that target the extracellular domain (ECD) of the receptors (Gala and Chandarlapaty 2014).

Eight tyrosine kinase inhibitors (TKIs), which bind actively to the kinase domain of HER family, have been approved for clinical use. The first generation of TKIs for HER family include erlotinib (Zhou et al., 2011), gefitinib (Mok et al., 2009; Han et al., 2012) and lapatinib (Xia et al., 2002; Schlam and Swain 2021). Erlotinib and gefitinib, which bind selectively to ATP-binding sites of EGFR, have been so far the only two single-target TKIs, whereas lapatinib, the first TKI approved for breast cancer shows equal activity toward EGFR and HER2. The next generation of TKIs including afatinib (Soria et al., 2015; Park et al., 2016; Paz-Ares et al., 2017), dacomitinib (Wu et al., 2017; Mok et al., 2018) and neratinib (Chan 2016; Chan et al., 2021) are irreversible pan-HER2 inhibitors (EGFR, HER2 and HER4), with afatinib and dacomitinib approved for non-small cell lung cancer (NSCLC) while neratinib approved for breast cancers. Among the above six TKIs, erlotinib, gefitinib and afatinib have currently remained the first-line treatments for NSCLC. Lastly, osimertinib has been known for a third-generation EGFR TKI, showing striking efficacies towards NSCLS patients with EGFR-activating mutations and EGFR T790M mutation (Mok et al., 2017; Remon et al., 2018; Papadimitrakopoulou et al., 2020). In general, TKIs against HER-family have evolved over the past 20 years; however, no patients can currently be cured with single treatment of TKI. Besides, the emergence of required resistance and the off-target toxicity associated with the treatment of TKI are the major challenges for the usage of TKIs in HER-driven cancers.

Different than TKIs, mAbs bind to the extracellular domains of the receptors, thus preventing the interaction of receptors with ligands or their dimerization partners (Hamilton et al., 2012). To date, multiple mAbs targeting HER receptors have been approved for clinical use. There are currently two HER2-targeting mAbs on the market, including trastuzumab (Herceptin^®^) and pertuzumab (Perjeta^®^). Trastuzumab was first approved in 1998 and has been widely used for the treatment of HER2-postive breast cancer and gastric cancer, while pertuzumab was approved in 2012 and has been used to treat HER2-positive breast cancer (Baselga et al., 2012) since 2012. Dual HER2 blockage with trastuzumab and pertuzumab has become the first-line treatment for patients with metastatic breast cancer (Swain et al., 2015). Up to date, there have been five EGFR-targeting mAbs approved for clinical uses, namely cetuximab (Erbitux^®^) (Lee et al., 2008), panitumumab (Vectibix^®^) (Gemmete and Mukherji 2011), nimotuzumab (BIOMAB-EGFR^®^), and necitumuma (Portrazza^®^) (Thatcher et al., 2015) and amivantamab (amivantamab-vmjw; Rybrevant^®^). Both cetuximab and panitumumab were approved for metastatic colorectal cancer (CRC), whereas cetuximab were also approved for the treatment of KARS wild-type CRC and advanced squamous cell cancer (Cai et al., 2020). Nimotuzumab were approved to treat head and neck squamous cell cancer, and it is also known as an orphan drug for gliomas (Subramanian et al., 2018; Koramati et al., 2021). Necitumumab was approved for the treatment of refractory metastatic squamous NSCLC (Thakur and Wozniak 2017). Different than the aforementioned anti-EGFR mAbs, amivantamab is a bispecific antibody that binds simultaneously to the extracellular domains of EGFR and MET, and has been recently approved for the treatment of adult patients with advanced or metastatic NSCLC with EGFR exon 20 insertion mutations (Syed 2021).

Overall, despite the significant advances resulted from the current approaches of targeted therapy, new therapeutics for HER-positive cancers remain in a high demand. Targeted therapies with TKIs or mAbs alone often shows inadequate efficacy, due to their low cytotoxicity and poor penetrance into tumors. With the rapid expanding of biopharmaceutical market, improved therapeutic options, such as ADCs, and the combination of ADCs with TKIs, mAbs, chemotherapeutics or immunoagents, are also extensively explored in many ongoing clinical trials.

### Antibody-Drug Conjugates Targeting EGFR

EGFR is amplified or overexpressed in a variety of tumor types, and has been validated as an important oncology target. However, there is no ADC approved for EGFR-based therapy. At present, three ADCs targeting ADCs are under clinical investigations, including depatuxizumab mafodotin (ABT-414), MRG003 and M1231 (Table 1). ABT-414 has been so far the most advanced investigational EGFR-driven ADC that has reached phase III trials.

**TABLE 1:** Summary of anti-EGFR ADCs in clinical investigation.

ADC	mAb	Payload	Linker	DAR	Conditions	Clinical phase	Company
ABT-414	Anti-EGFR mAb (ABT-806)	MMAF	Non-cleavable mc linker	4	Glioblastoma	II/III	AbbVie
MRG003	Anti-EGFR mAb	MMAE	Cleavable vc linker	n/a	Biliary tract cancer, nasopharyngeal carcinoma, squamous cell carcinoma of head and neck, non-small cell lung cancer	Ⅱ	Miracogen
M1231	Bispecific antibody that targets MUC1 and EGFR	Hemiasterlin	n/a	n/a	Metastatic solid tumors, esophageal cancer, non-small cell lung cancer	I	Sutro, EMD Serono

ADC: antibody-drug conjugates; DAR: drug-to-antibody ratio; mAb: monoclonal antibody; mc: maleimidocaproyl; MMAE: monomethyl auristatin E; MMAF: monomethyl auristatin F; vc: valine-citrulline.

#### Depatuxizumab Mafodotin (ABT-414)

Depatuxizumab Mafodotin (ABT-414) is composed of a EGFR-specific humanized antibody (ABT-806), a non-cleavable maleimidocaproyl (mc) linker and monomethyl auristatin F (MMAF), which inhibits microtubule assembling (Phillips et al., 2016; Van Den Bent et al., 2020). The average number of MMAF conjugated to each mAb is approximately 4 (Goss et al., 2018). ABT-806 binds to a cryptic epitope in a cysteine-rich domain (CR1) of EGFR that only opens up for antibody binding with EGFR amplified or overexpressed, or with the presence of mutant EGFR variant III (von Achenbach et al., 2020). Compared to other EGFR-targeting antibodies, including cetuximab and panitumumab, ABT-806 displayed minimal binding activity to EGFR in normal tissue, and was well tolerated at a relatively high dose (24 mg/kg) in a phase I clinical study (Phillips et al., 2016). ABT-414 was found to retain the excellent binding and functional properties of ABT-806, and exhibit significant efficacy against glioblastoma patient-derived xenograft models with either wild-type EGFR or mutant EGFR overexpression (van den Bent et al., 2017). A phase I clinical trial (NCT01800695) was carried out to determine the safety, pharmacokinetics and antitumor efficacy of ABT-414 as a mono-therapy or in combination with temozolomide (an alkylating agent used for glioblastoma multiforme) in glioblastoma patients. ABT-414 demonstrated manageable safety and acceptable pharmacokinetic profiles in phase I trial. However, the following phase II study (NCT02343406) did not meet primary endpoint of overall survival. Moreover, ABT-414 showed no impact on the health-related quality of life and neurological deterioration-free survival in EGFR-amplified recurrent glioblastoma, except for irreversible ocular toxicity, an expected side effect from MMAF (Phillips et al., 2018). A phase II/III study (NCT02573324) was currently underway in participants with newly diagnosed glioblastoma with EGFR amplification. However, enrollment in this trial has been halted since 2019 due to lack of survival benefit for patients receiving ABT-414 (NCT02573324).

#### MRG003

MRG003 is composed of a fully human EGFR-specific IgG1 antibody, a protease cleavable valine-citrulline (vc) linker and monomethyl auristatin E (MMAE). The preclinical data of MRG003 has not been published. Phase I clinical trials (CTR20180310, NCT04868344) have been conducted to evaluate the safety, pharmacokinetics and efficacy of MRG003 as a single agent in patients with relapsed or refractory solid tumors, including colorectal, nasopharyngeal, head and neck, esophageal, and duodenal cancer (Xu et al., 2020). Encouragingly, MRG003 has demonstrated acceptable safety profiles and potential antitumor activity. Currently, a number of phase II studies of MRG003 (NCT05126719, NCT04868162, NCT04838964 and NCT04838548) are under way in patients with recurrent or metastatic nasopharyngeal carcinoma, squamous cell carcinoma of head and neck, advanced metastatic biliary tract cancer and advanced NSCLC, respectively.

#### M1231

M1231 is an investigational ADC with conjugation of a hemiasterlin-related payload to a bispecific antibody that targets MUC1 and EGFR simultaneously. Hemiasterlin is a tripeptide that exerts its cytotoxicity through binding to tubulin, thus disrupting normal microtubule dynamics. The detailed structural information and preclinical data of M1231 have not yet been published. M1231 is currently under a phase I investigation as a monotherapy in patients with metastatic solid tumors, esophageal cancer and NSCLC (NCT04695847).

### Antibody-Drug Conjugates Targeting HER2

**FIGURE 2:**
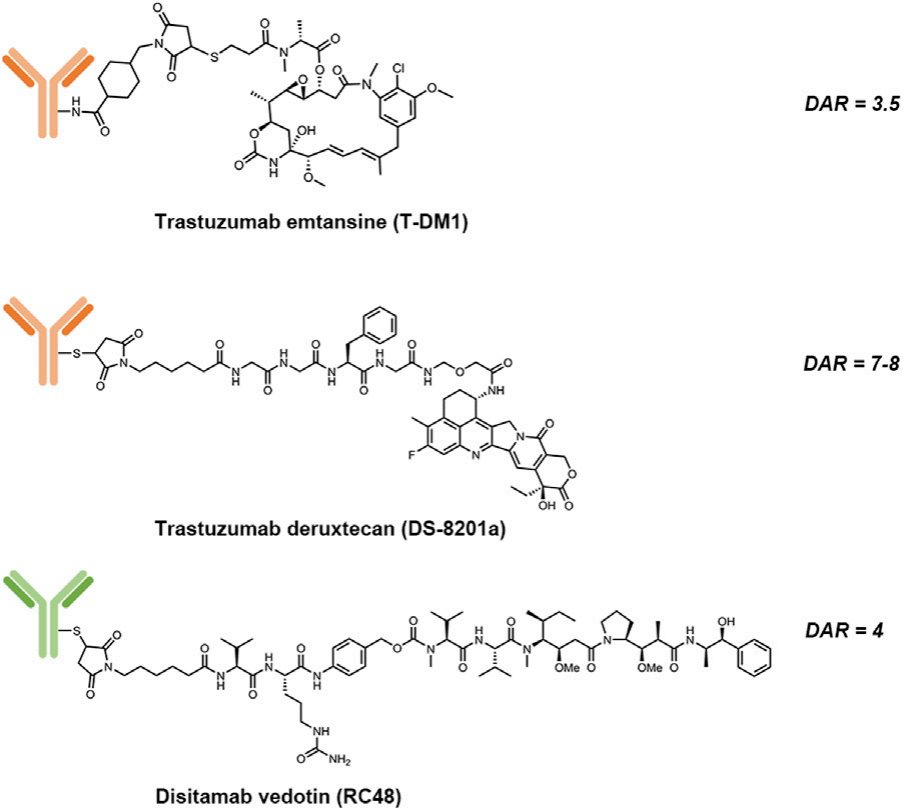
Structures of trastuzumab emtansine (T-DM1), trastuzumab deruxtecan (DS-8201a) and disitamab vedotin (RC-48).

HER2 is another established target from the HER family for cancer therapies. Up to date, there have been three HER2-directed ADCs on the market, which were either approved by the U.S. Food and Drug Administration (FDA) or the National Medical Products Administration (NMPA) of China (Table 2). The structures of these three approved ADCs are shown in Figure 2. Seven HER2-directed ADCs are currently under clinical investigations. An overview of these ADCs, including their parent antibodies, linkers, drugs, conditions and clinical trial status, are summarized in Table 3.

**TABLE 2:** Currently approved anti-HER2 ADCs on the market.

ADC	Trade Name	mAb	Payload	Linker	DAR	Approved Indications	Company	Approval Agency	Approval Year
Trastuzumab emtansine (T-DM1)	Kadcyla	Trastuzumab	DM1	Non-cleavable SMCC linker	3.5	Metastatic HER2-positive breast cancer	Genentech	U.S. FDA	2013
Trastuzumab deruxtecan (DS-8201a)	Enhertu	Trastuzumab	Dxd	Cleavable GGFG linker	7–8	Metastatic HER2-positive breast cancer, locally advanced or metastatic HER2-positive gastric or gastroesophageal adenocarcinoma	Daiichi Sankyo	U.S. FDA	2019 (breast cancer); 2021 (gastric or gastroesophagel cancer)
Disitamab vedotin (RC48)	Aidixi	Hertuzumab	MMAE	Cleavable vc-PABC linker	4	Locally advanced or metastatic HER2-positive gastric or gastroesophageal cancer	RemeGen	China NMPA	2021

ADC: antibody-drug conjugates; DAR: drug-to-antibody ratio; FDA: food and drug administration; GGFG: glycine-glycine-phenylalanine-glycine; NMPA: national medical products administration; SMCC: N-succinimidyl-4-(N-maleimidomethyl) cyclohexanecarboxylate; vc-PABC: valyl-citrullinyl-*p*-aminobenzyloxycarbonyl.

**TABLE 3:** Summary of anti-HER2 ADCs in clinical investigation.

ADC	mAb	Payload	Linker	DAR	Conditions	Clinical phase	Company
Trastuzumab duocarmazine (SYD985)	Trastuzumab	seco-DUBA	Cleavable vc linker	2.7	Breast cancer, endometrial cancer	Ⅰ (solid tumors, HER2-posive, HER2-low and metastatic breast cancer)	Byondis
						I/Ⅱ (metastatic breast cancer)	
						Ⅱ (endometrial cancer)	
ARX-788	Anti-HER2 mAb (ARX269)	MMAF	Non-cleavable linker conjugated to pAcF	1.9	Breast cancer, gastric cancer	I (breast neoplasms, gastric neoplasm, solid tumors)	Ambrx
						Ⅱ (metastatic breast cancer, breast and gastric neoplasm cancer, breast cancer with low expression of HER2)	
A166	Anti-HER2 mAb	Duostatin-5	Cleavable vc linker	n/a	Solid tumors expressing HER2 or having amplified HER2 gene	Ⅰ/Ⅱ	Klus
MRG002	Anti-HER2 mAb	MMAE	Cleavable vc linker	3.8	Breast cancer, gastric cancer, gastroesophageal junction cancer, non-small cell lung cancer, urothelial cancer, biliary tract cancer	Ⅰ (advanced solid tumor)	Miracogen
						Ⅱ (locally advanced gastric cancer and metastatic gastroesophageal junction cancer, non-small cell lung cancer, advanced or metastatic breast cancer, locally advanced or metastatic urothelial cancer, advanced or metastatic biliary tract cancer)	
ALT-P7	Trastuzumab biobetter (HM2)	MMAE	Cleavable cysteine-containing peptide linker	2	Breast cancer	I	Alteogen
GQ1001	Trastuzumab	DM1	n/a	n/a	Breast cancer, gastric cancer, advanced solid tumor	I	GeneQuantum
SBT6050	Anti-HER2 mAb	Toll-like receptor 8 agonist	n/a	n/a	Breast cancer, gastric cancer, colorectal cancer, non-small cell lung cancer	I (solid tumors), Ⅱ (breast cancer, gastric cancer, colorectal cancer, non-small cell lung cancer)	Silverback

ADC: antibody-drug conjugates; DAR: drug-to-antibody ratio; mAb: monoclonal antibody; pAcF: *para*-acetylphenylalanine; seco-DUBA: seco-duocarmycin-hydroxybenzamide-azaindole.

#### Trastuzumab Emtansine

Trastuzumab emtansine (T-DM1) is composed of: 1) trastuzumab, a humanized HER2-targeting mAb that have been approved for the treatment of HER2-positive breast cancer; 2) a non-cleavable thioether linker, N-maleimidomethyl cyclohexane-1-carboxylate (MCC); and 3) a potent microtubule-depolymerizing maytansinoid derivative, DM1 (Corrigan et al., 2014). An average of 3.5 molecules of DM1 were conjugated to lysine residues on trastuzumab. The mechanism of action of T-DM1 are believed to be involved with the functions of both trastuzumab and DM1, which include trastuzumab-mediated inhibition of HER2 signaling, induction of antibody-dependent cell-mediated cytotoxicity (ADCC) by the Fc domain of trastuzumab, and the release of DM1 metabolites that interrupt with microtubule aggregation and cause cell apoptosis (Costa and Czerniecki 2020). T-DM1 was first approved by the U.S. FDA as a single agent for the treatment of metastatic HER2-positive breast cancer in 2013. Currently, there are more than 100 clinical trials underway to evaluate either the use of T-DM1 for other HER2-driven cancer types or combination therapies of T-DM1 with other agents, such as immune checkpoint inhibitors, CDK4/6 inhibitors and TKIs.

#### Trastuzumab Deruxtecan

Trastuzumab deruxtecan (T-Dxd; DS-8201a) is the second approved HER2-targeting ADCs. The components of DS-8201a are: 1) trastuzumab; 2) an enzymatically cleavable maleimide glycine-glycine-phenylalanine-glycine (GGFG) peptide linker that can be cleaved by lysosomal proteases while maintaining stable in serum; and 3) a topoisomerase I inhibitor DXd, which is a novel water-soluble derivative of exatecan, a hexacyclic camptothecin analogue (Xu H et al., 2019). Through binding to topoisomerase I-DNA complex, Dxd induces double-strand DNA damage and cell apoptosis. About 7 to 8 Dxd molecules are conjugated to the cysteine residues on trastuzumab. Preclinical studies showed that DS-8201a possessed a higher antitumor activity than T-DM1, including efficacy against HER2-low tumors (Ogitani et al., 2016). The improved efficacy of DS-8201a may result from its higher drug-to-antibody ratio (DAR), the cleavable GGFG peptide linker and the better membrane permeability of Dxd payloads, which together lead to a stronger bystander effect on non-targeted cancer cells (Xu Z et al., 2019).

DS-8201a was approved by the U.S. FDA for patients with metastatic HER2-positive breast cancer in 2019. The approval was based on the finding from a phase II clinical trial (NCT03248492), which showed that DS-8201a exert durable antitumor activity in patients who had already received treatment with T-DM1 for their metastatic or unresectable HER2-positive breast cancer and the recommended dose is 5.4 mg/kg given once every 3 weeks (Modi et al., 2020). In 2021, the U.S. FDA has also approved DS-8201a for the treatment of patients with advanced or metastatic gastric or gastroesophageal junction adenocarcinoma. This approval was based on the assessment of a phase II trial (NCT03329690) among patients with HER2-positive advanced gastric or gastroesophageal junction cancer. In this clinical study, DS-2108a demonstrated notable antitumor activity and significantly improved objective response rate and overall survival in patients compared to conventional chemotherapy (Shitara et al., 2020). Currently, around 40 clinical trials have evaluated or are investigating the effects of DS-8201a as a monotherapy or combination therapy on patients with a variety of HER2-positive cancers.

#### Disitamab Vedotin

Disitamab vedotin (RC48) is a novel ADC comprised of a humanized HER2-specific mAb (hertuzumab) linked to the cytotoxic MMAE via a maleimdocaproyl-valyl-citrullinyl-*p*-aminobenzyloxycarbonyl (mc-val-cit-PABC) linker, with a DAR value of 4 (Yao et al., 2015). Vedotin, referring to MMAE and the mc-val-cit-PABC linker, has already been validated for an approved ADC, brentuximab vedotin (Adcetris^®^) (Chari et al., 2014). Compared to trastuzumab, hertuzumab possesses a higher HER2-specific affinity and shows greater ADCC activity *in vitro* (Li H et al., 2016). With a cleavable linker, RC48 was shown to exhibit significant bystander effects where the payloads diffused to adjacent cells, which was not the case with T-DM1. In addition, RC48 showed superior antitumor activity than T-DM1 not only in HER2-overexpressing xenograft tumor models but also in trastuzumab- and lapatinib-resistant xenograft tumor models (Yao et al., 2015; Li H et al., 2016).

A phase I study (NCT02881190) of single agent of RC48 was conducted in patients with advanced or metastatic HER2-positive solid carcinomas, which showed that RC48 had tolerable toxicity and substantial potency against HER2-positive solid tumors, especially in HER2-low expression gastric cancer (Xu et al., 2021). Additionally, a phase II study (NCT03556345) of RC48 in patients with advanced or metastatic HER2-positive gastric or gastroesophageal junction cancer demonstrate a 24.8% objective response rate, a median progression-free survival of 4.1 months, and a median overall survival of 7.9 months (Peng et al., 2021). Based on the results of this study, RC48 was granted conditional marketing approval by the NMPA of China for the treatment of patients with locally advanced or metastatic gastric or gastroesophageal junction cancer who have received at least two types of chemotherapy in June 2021. Concurrently, a number of early- or late-stage clinical trials are underway in patients with multiple solid tumor types, including urothelial cancer, breast cancer, gynecological malignancy and NSCLC.

#### Trastuzumab Duocarmazine

Trastuzumab duocarmazine (SYD985) is a novel ADC composed of the anti-HER2 trastuzumab, a cleavable valine-citrulline peptide linker, and a duocarmycin derivative, which is present inactive as seco-duocarmycin-hydroxybenzamide-azaindole (sec-DUBA) (Elgersma et al., 2015). Once the seco-DUBA is activated by proteases, the active duocarmycin is released, which can bind to the minor groove of the DNA, causing irreversible alkylation of DNA and eventually cell death (Dokter et al., 2014). In addition, the membrane-permeable duocarmycin can further induce a significant bystander killing effect, thus providing a wide therapeutic window. Compared to T-DM1, SYD985 was shown to be active in HER-low breast cancer xenograft models (van der Lee et al., 2015). The result of phase I clinical trial (NCT02512237) confirmed the antitumor effect of SYD985, although the ocular adverse reaction was commonly reported (Menderes et al., 2017; Banerji et al., 2019). A phase III randomized control trial (NCT03262935) is currently underway in patients with HER2-positive locally advanced or metastatic breast cancer.

#### ARX-788

ARX-788 is a novel ADC composed of an anti-HER2 mAb, a non-cleavable linker and a proprietary version of MMAF (Amberstatin 269 or AS269). The payload was site-specifically conjugated to a *para*-acetylphenylalamine (pAcF), a non-natural amino acid that is incorporated into a defined position on the heavy chain with a DAR around 1.9 (Abdollahpour-Alitappeh et al., 2019). ARX-788 was found to be more effective than T-DM1 in a breast cancer xenograft model resistant to trastuzumab (Barok et al., 2020). Moreover, AX788 can eliminate tumor in breast cancer and gastric cancer that are resistant to T-DM1 (Barok et al., 2020). ARX-788 is currently under investigation in two phase I clinical trials (NCT02512237 and NCT03255070). A variety of phase II clinical trials are underway to study the role of ARX-788 in HER2-positive metastatic breast cancer (NCT05018676), selected HER2-mutated or HER2-amplified solid tumors (NCT05041972), HER2-low breast cancers (NCT05018676) and HER2-positive breast cancer with brain metastasis (NCT05018702).

#### A166

A166 is composed of an anti-HER2 antibody and a highly potent MMAF-derived payload (duostatin-5) via a cleavable valine-citrulline linker (Liu et al., 2020). A phase I/II clinical trial showed that A166 is clinically effective in patients with relapsed or advanced solid tumors. Responses were observed at the dose level of 3.6 mg/kg and 4.8 mg/kg, and an objective response rate of 36% was achieved at efficacious dose level (NCT03602079).

#### MRG002

MRG002 is composed of a humanized anti-HER2 IgG1 mAb, a valine-citrulline linker and the microtubule disrupting MMAE. The average DAR is 3.8 (Li et al., 2021). In preclinical study, MRG002 demonstrated potent antitumor activities in the breast and gastric patient-derived xenograft models with varying levels of HER2 expression. MRG002 also showed superior potency than trastuzumab and T-DM1 in mouse xenograft models. Moreover, a combination of MRG002 with anti-PD-1 antibody was found to significantly enhance antitumor activity. Phase I studies of MRG002 as a single agent is underway in patients with relapsed/refractory solid tumors, including breast cancer, gastric cancer, salivary gland cancer (CTR20181778 and NCT04941339). Concurrently, a variety of phase II trials are studying the efficacy of MRG002 in multiple HER2-positive or HER2-low malignancies.

#### ALT-P7

The novel ADC ALT-P7 (HM2-MMAE) is comprised of trastuzumab biobetter HM2 and the toxin payload MMAE through site-specific cysteine conjugation (Rinnerthaler et al., 2019). ALT-P7 is currently under investigation in phase I clinical trial with HER2-positive breast cancer patients (NCT03281824). ALT-P7 demonstrated an acceptable safety profile with dose limiting toxicities observed at 4.8 mg/kg and 4.5 mg/kg under evaluation, which warrants further investigation in a phase II trial (Yeon et al., 2020).

### Antibody-Drug Conjugates Targeting HER3

HER3 is overexpressed in a variety of cancer types, and has been suggested to predict poor prognosis. Despite lacking significant kinase activity, HER3 exerts its function through HER3 homodimerization or HER2/HER3 heterodimerization, thus activating downdream signaling pathways to promote cell survival and proliferation (Wallasch et al., 1995; Sierke et al., 1997; Berger et al., 2004; Shi et al., 2010). Importantly, HER3 signaling has been shown to be associated with the resistance mechanism of anti-EGFR/HER2 therapies (Erjala et al., 2006; Engelman et al., 2007; Sergina et al., 2007; Yonesaka et al., 2019), and is emerging as a promising therapeutic target for EGFR-mutant NSCLC. Patritumab deruxtecan (U3-1402) is the only one ADC that is currently under clinical investigation.

#### U3-1402

Patritumab deruxtecan (HER3-Dxd; U3-1402) is composed of an anti-HER3 mAb (patritumab), a cleavable GGFG linker, and the topoisomerase I inhibitor DXd (Haratani et al., 2020). The DAR of U3-1402 is 8. U3-1402 displayed a high HER3-specific binding affinity among other human HER family receptors, including EGFR, HER2 and HER4, and was also shown to possess potent antitumor activity in patient-derived xenograft models with an acceptable safety profile (Hashimoto et al., 2019). A mechanism of action study showed that the activity of U3-1402 was driven by an efficient internalization and intracellular trafficking of ADCs to lysosome to release the toxic compounds (Koganemaru et al., 2019). Also, significant tumor regression with the treatment of U3-1402 was observed in the colorectal tumor xenograft models. In addition, the administration of U3-1402 alone or in combination with an EGFR-TKI was shown to significantly suppress the growth of EGFR-TKI-resistant NSCLC xenograft tumors (Yonesaka et al., 2019). A phase I/II study (NCT02980341) of U3-1402 is underway in HER3-positive metastatic breast cancer. An early report of this trial suggested that U3-1402 possessed promising antitumor activity with a tolerable safety profile (Schoffel et al., 2016; Yonesaka 2021). Concurrently, a phase I clinical study is ongoing to study U3-1402 in metastatic or unresectable NSCLC (NCT03260491).

### Summary and Future Directions

HER-directed ADCs are emerging as a highly promising therapeutic for patients with HER-positive cancers, with three anti-HER2 ADCs (T-DM1, DS-8201a and RC48) approved for HER2-positive cancer and 11 HER-directed ADCs currently in clinical trials. Three EGFR-directed ADCs (ABT-414, MRG003 and M1231) have entered clinical trials with advanced EGFR-expressing malignancies. A total of seven HER2-directed ADCs (SYD985, ARX-788, A166, MRG002, ALT-P7, GQ1001 and SBT6050) are currently being investigated in clinical trials, among which SYD985 and ARX-788 showed greater potency in HER2-low breast cancer than T-DM1. There is only one single HER3-directed ADC (U3-1402) that is currently under clinical study, which has demonstrated promising results in patients with HER3-positive metastatic breast cancer and metastatic EGFR-mutant NSCLC. Noteworthily, the first biosimilar of T-DM1, Ujvira^®^, was launched by Zydus Cadila in India in 2021 for treating both early and advanced HER2-positive breast cancer.

As one of the fastest growing anticancer drugs, ADCs are currently facing three major challenges: 1) How to improve cancer cell uptakes of ADCs has been the major challenge associated with the development of ADCs. At present, ADCs rely on high expression level of target antigen on the surface of cancer cells to ensure effective endocytosis to release cytotoxic payloads. Studies have shown that effective cell killing by HER2-targeting ADC was in general correlated to the level of HER2 expression on the cell surfaces (Li J. Y et al., 2016), and typically required a rather high level of surface HER2 expression (∼10^6^ surface receptors per cell) (Andreev et al., 2017). The expression level of target antigen on tumor surfaces has been significantly limiting the therapeutic efficacy of the existing ADCs. Therefore, improving cancer cell uptakes of ADCs could potentially address the market needs, especially for patients with lower antigen expression level. It is noteworthy that the major proportion of patients of breast cancers (40–50%) are categorized as HER2-low (Eiger et al., 2021), so a treatment option for such a large population would be highly demanded. 2) Systematic toxicity remains one of the main factors that contribute to the failure of ADC clinical trials. The toxic effect has been linked to diverse factors, including the antibody, the payload drug, the linker and the target antigen. Lack of ADC internalization (Donaghy 2016), non-specific binding of antibodies to Fc receptors (Donaghy 2016), early cleavage of linkers to release free drugs (Agatsuma 2017), or bystander effect caused by super-cytotoxic payload to normal cells (Staudacher and Brown 2017) may induce toxic effects to non-target cells. Also, low expression of target receptors in normal tissues is another important factor that leads to off-target toxicity of ADCs (Xu 2015). 3) The emerging resistance to ADC treatment is another hurdle to overcome. Studies have indicated that ineffective internalization and lysosomal trafficking or degradation of ADCs could be the major mechanism of resistance to T-DM1 (Takegawa et al., 2017; García-Alonso et al., 2020; Díaz-Rodríguez et al., 2022). Required resistance to the free cytotoxic drug (DM1) through upregulation of drug efflux pumps or alternation of tubulins/microtubule-associated proteins, could also be responsible for resistance to T-DM1 (García-Alonso et al., 2020). In addition, mechanisms of resistance related to antibody (trastuzumab) may also contribute to T-DM1 resistance, including decreased expression of HER2, expression of truncated forms of HER2, or mutations in the ERBB2 gene (Bon et al., 2020; Díaz-Rodríguez et al., 2022).

The possible future directions for ADC development to conquer the above challenges may include: 1) Recombinant antibody approaches may be explored to improve cancer cellular delivery and lysosomal trafficking of ADCs. At present, a variety of dual antibodies, including bispecific antibodies and biparatopic antibodies are intensively investigated to increase ADC internalization and lysosomal delivery (Hosseini et al., 2021). Alternative antibody engineering strategies, such as antibody recombination with lysosome-sorting peptides or cell-penetrating peptides, have also been explored to improve cancer targeting and lysosomal delivery of ADC (Han et al., 2020). 2) Improvements in ADC design remain in high demand. In the next generation of ADCs, it is necessary to develop new payload platforms, linker technologies and conjugation strategies to maximize the therapeutic efficacy and minimize the toxicity of ADCs. New drug scaffolds with higher efficacies, fewer side effects and different mechanisms of action are driving the next generation of ADCs into the market (Singh et al., 2015; Pettinato 2021). Novel technologies for designing cleavable linkers and engineering the releasing mechanism for them will continue to be an important future direction (Leung et al., 2020, Xu et al., 2019). Significant efforts are still being directed towards the development of effective site-specific conjugation methods to ensure the production of homogeneous ADCs with consistent quality (Yamada and Ito 2019). 3) Clinical and translational approaches will also play a critical role in improving the therapeutic window of ADCs. Combination therapies are thought to possess the ability to improve drug efficacy and reduce drug resistance of ADCs (Ge et al., 2017; Coats et al., 2019). In addition, clinical biomarkers to optimize patient selection and monitor response signals are also required to improve therapeutic index of ADCs (Coats et al., 2019).

Above all, ADCs have created a new paradigm for cancer therapy, and will continue to represent a unique and powerful therapeutic approach through decreasing systematic toxicity, enhancing therapeutic efficacy and reducing drug resistance.
